# Fast Specimen Boundary Tracking and Local Imaging with Scanning Probe Microscopy

**DOI:** 10.1155/2018/3979576

**Published:** 2018-03-05

**Authors:** Yongbing Wen, Jianmin Song, Xinjian Fan, Danish Hussain, Hao Zhang, Hui Xie

**Affiliations:** State Key Laboratory of Robotics and Systems, Harbin Institute of Technology, Harbin 150080, China

## Abstract

An efficient and adaptive boundary tracking method is developed to confine area of interest for high-efficiency local scanning. By using a boundary point determination criterion, the scanning tip is steered with a sinusoidal waveform while estimating azimuth angle and radius ratio of each boundary point to accurately track the boundary of targets. A local scan region and path are subsequently planned based on the prior knowledge of boundary tracking to reduce the scan time. Boundary tracking and local scanning methods have great potential not only for fast dimension measurement but also for sample surface topography and physical characterization, with only scanning region of interest. The performance of the proposed methods was verified by using the alternate current mode scanning ion-conductance microscopy, tapping, and PeakForce modulation atomic force microscopy. Experimental results of single/multitarget boundary tracking and local scanning of target structures with complex boundaries demonstrate the flexibility and validity of the proposed method.

## 1. Introduction

Scanning probe microscopes (SPMs) [1] are commonly used in the field of nanoscience and technology for high-resolution imaging and quantitative measurements of nanoscale properties. Atomic force microscopy (AFM) [2] and scanning ion-conductance microscopy (SICM) [3] are well-established SPM techniques. In SPM, a probe with a very sharp tip is utilized to detect the sample surface. Generally, the probe or sample is moved to follow a predefined raster scan trajectory in* xy*-plane with the tip-sample distance controlled on the *z*-axis for imaging the specimen surface, which is a time-consuming process. Therefore, significant research efforts have been invested to minimize the scanning time by either improving SPM hardware or scanning algorithms, for example, specially designed optical beam deflection setup [4, 5], innovative mechanical body design [6, 7], high resonance frequency [8–11] or wide-area [12] nanopositioners, advanced modern control techniques, such as feedforward/feedback controller [13–15], active damping algorithm [16], and dynamic proportional-integral-differential controller [17], for the piezo actuators to eliminate the mechanical resonant vibrations in high-speed imaging. On the other hand, several interesting methods are developed for high-speed scanning with smooth scanning trajectories without modification in hardware such as sinusoidal waveform [18, 19], spiral scanning [20, 21], cycloid-like scanning [22], and Lissajous scanning paths [23, 24].

However, one significant similarity in the above-mentioned approaches is the larger subregion outside the region of interest during scanning, which severely limits the scanning performance. In contrast, a series of local scanning methods [25–28] for imaging the desired target regions have been developed. Andersson et al. [26, 27] proposed a high-level feedback control approach for rapidly imaging the sample with string-like boundaries. This method is designed for tracking the edge of the object to reduce the overall imaging time by reducing the total sampling area. Zhang et al. [28] presented a novel scanning method for specimens of fairly simple structure, where its contours are smooth. Sample topography is constructed from the surface edge at different height without crossing the specimen during scanning process to minimal interaction with the specimen.

In this work, we focus on local scanning of the target by reducing the overall scanning area to minimize the imaging time. A boundary tracking algorithm is used to locate the target of interest without entering its interior according to the view of optical microscopy. A local scan area is determined by combining the moving least squares method with a mathematical analytical method. At last, the corresponding scan path is planned to get the topography of the target. The applicability and versatility of the method are confirmed with the experimental results by using SICM and AFM. In order to extend the application of the proposed method in the field of biological sciences [29], a bacterial cell was scanned to obtain the physical properties of the surface. This method has several promising potential applications, for example, automatically tracking and scanning the scattered targets with the optical guiding and path planning, tracking the shape of cellular motility or growth, and investigating the self-assembly processes of structures.

## 2. Boundary Tracking and Local Scanning

The boundary tracking and local scanning are accomplished in three steps. Firstly, criteria for the boundary point tracking are defined and the boundary of the target structure is determined by using the criteria defined in the first step. In case of sharp corners or edges, a sinusoidal wave cannot track the boundary of the target structure. Therefore, an additional step of lost-step processing and adaptive step size adjustment is used to correct the trajectory of the probe for tracking the sample boundary correctly. Secondly, the edge of the target is locally fitted with the moving least squares method (MLS) according to the obtained boundary points. An analytical method is adopted to obtain the discrete points on the equidistant curve with downsampling subsequently. Finally, the local scanning algorithm is initiated for imaging the target topography.

### 2.1. Boundary Tracking

**Figure 1:**
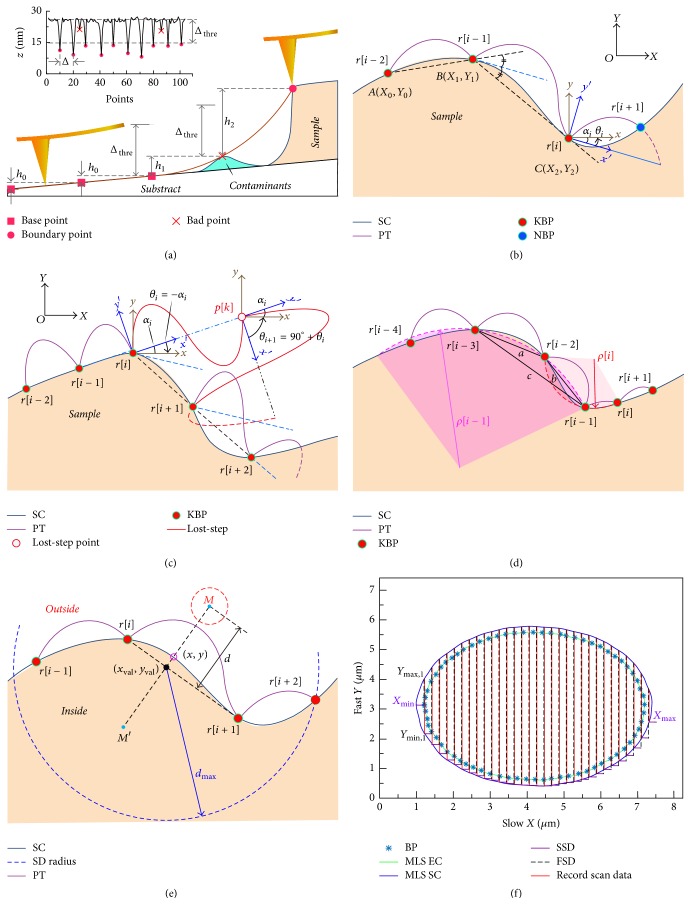
Illustration of boundary tracking and local scanning method. Schematic of boundary point determination criteria (a), azimuth angle prediction (b), lost-step (c), adaptive step size adjustment (d), moving least squares (MLS) method (e), and planning local scanning path (f).

Firstly, boundary point determination criterion (BPDC) for searching boundary points based on the height difference of adjacent sampling points is defined. The BPDC is a function that determines whether the current point is a boundary point or not. A schematic diagram of the BPDC function is shown in Figure 1(a), where* h*_0_ represents the height difference between adjacent sampling points due to the sample inclination and* h*_1_ indicates the height difference between adjacent sampling points due to the contaminants in the vicinity of the target. The height* h*_2_ is regarded as the real boundary point when BPDC is satisfied. A proportional-integral-differential (PID) feedback controller is used to adjust the probe to follow the height variations of the sample contour (SC). By recording the *z*-position of the probe, the boundary point can be recognized with the difference of the current (*Ps*_*i*,*j*_) and previous (*Ps*_*i*,*j*−1_)* z*-position and a set threshold (Δ_thre_). So, a decision function *H*(*i*, *j*) of boundary point can be given as

(1) $$\begin{array}{c} H\left(\;i,j\;\right)=\left\{\begin{array}{cc} 1, & \left|{Ps}_{i,j}-{Ps}_{i,j-1}\right|>\Delta_{thre}\\ 0, & else, \end{array}\right. \end{array}$$

where* i* is the searched* i*th boundary point,* j* is the* j*th discrete sample point in a sinusoidal waveform cycle,* s*_i,j_ is the *z*-position of the* j*th discrete sampling point in the* i*th sinusoidal waveform, and* Ps*_i,j_ is the* z*-position of the probe which represents the surface topography. For the target features that are lower than the substrate (e.g., microhole), consecutive boundary point is determined by (*Ps*_*i*,*j*−1_−*Ps*_*i*,*j*_).

The tip is steered with a sinusoidal waveform with the current boundary point (*r*_i_) as the starting point. Once the next boundary point (*r*_i+1_) is detected, it is used as the starting point of the next sinusoidal cycle. This iteration is repeated to detect all the boundary points. As shown in Figure 1(b), a coordinate transformation of the three coordinate systems is introduced to determine the trajectory of probe (PT). Assume that the global coordinate system (GCS) *O*(*X*, *Y*) is fixed on the scanner. The coordinates of the known boundary point (KPB)* r*_i-2_,* r*_i-1_, and* r*_i_ in GCS are* A*(*X*_0_, *Y*_0_),* B*(*X*_1_, *Y*_1_), and* C*(*X*_2_, *Y*_2_), respectively. A relative coordinate system (RCS) *o*(*x*, *y*) is consistent with GCS and its origin is located at* r*_i_. The origin of the local coordinate system (LCS) *o*′(*x*′, *y*′) is also located at* r*_i_, and its *x*′-axis direction is consistent with the motion of* r*_i_. The position of each discrete sample point in LCS can be given as

(2) x′=Δd×j,y′=Aml×sin⁡2πjn,

where Δ_*d*_ is the distance between arbitrary adjacent discrete samples on the *x*′-axis and Aml is the tracking amplitude of the sinusoidal waveform which is divided into* n* parts along the *x*′-axis.

A vector $\vec{\xi }$ is defined as

(3) $$\begin{array}{c} \vec{\xi }=\left\{\begin{array}{cc} \frac{\vec{AB}}{\left|\vec{AB}\right|}+\frac{\vec{BC}}{\left|\vec{BC}\right|}, & \cos\langle \vec{AB},\vec{BC}\rangle >0\\ -\frac{\vec{AB}}{\left|\vec{AB}\right|}+\frac{\vec{BC}}{\left|\vec{BC}\right|}, & \cos\langle \vec{AB},\vec{BC}\rangle <0. \end{array}\right. \end{array}$$

The azimuth angle *α* can be calculated by $\vec{\xi }\cdot \vec{n_{X}}=\left|\vec{\xi }\right|\left|\vec{n_{X}}\right|\cos\alpha$ ($\vec{n_{X}}$ is the unit vector along the *x*-axis of GCS). Hence, $\alpha =\arccos\left(\vec{\xi }\cdot \vec{n_{X}}/(\left|\vec{\xi }\right|\left|\vec{n_{X}}\right|)\right)$.

**Figure 2:**
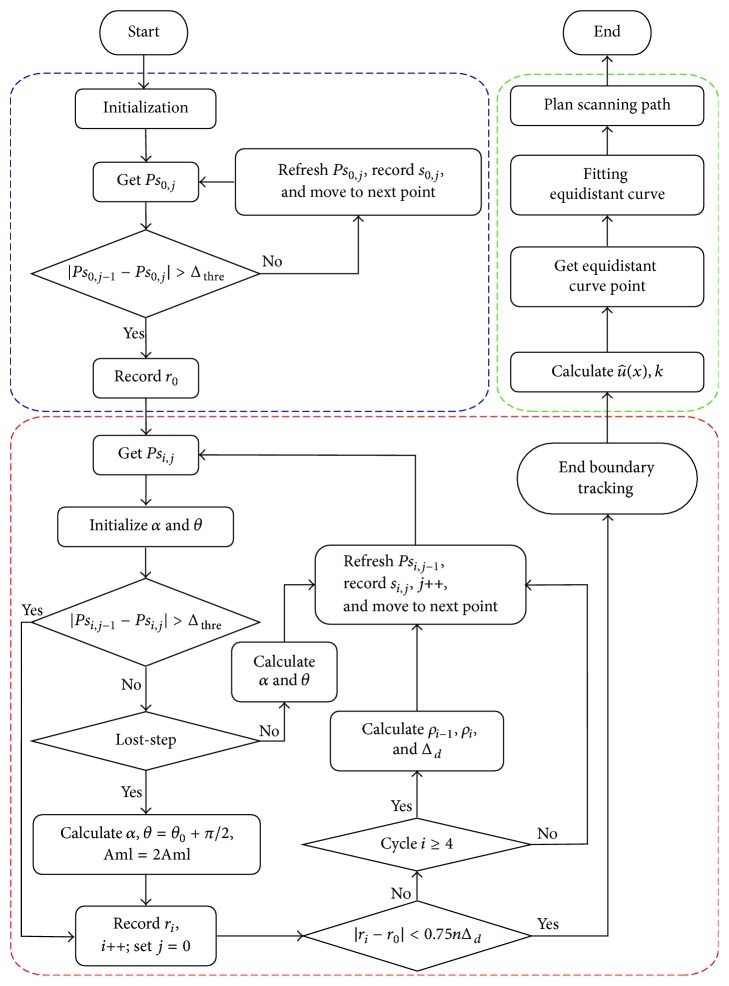
The flowchart of the boundary tracking algorithm and the local scanning.

Particularly, after correctly searching for the first boundary point (as shown in the blue dashed box in Figure 2), *α*_0_ should be chosen carefully to prevent the* lost-step* phenomenon as described in Section 2.2. Since the trajectory of the probe is set to the left side of the target structure as the starting position (clockwise), the corresponding *α*_0_ is usually selected as 90° (or 45°), and *θ*_0_ = −*α*_0_. For the second boundary point,*α*_1_ is determined by the first and second boundary points via $\alpha_{1}=arccos(\vec{BC}\cdot \vec{n_{X}}/(\left|\vec{BC}\right|\left|\vec{n_{X}}\right|))$.

The coordinates (*x*,* y*) in RCS can be expressed as

(4) $$\begin{array}{c} \left[\begin{array}{c} x\\ y \end{array}\right]=\left[\begin{array}{cc} \cos\theta & \sin\theta \\ -\sin\theta & \cos\theta \end{array}\right]\left[\begin{array}{c} x^{\prime }\\ y^{\prime } \end{array}\right], \end{array}$$

where*θ* is an angle from the *x*′-axis to the* x*-axis (counterclockwise):

(5) $$\begin{array}{c} \theta =\left\{\begin{array}{cc} \alpha , & Y_{2}<Y_{1}\\ -\alpha , & else. \end{array}\right. \end{array}$$

Thus, the coordinates (*X*, *Y*) of the next boundary point (NBP) can be predicted according to the KBP:

(6) X=X2+jΔdcos⁡θ+Aml×sin⁡2πjn⁡sin⁡θ,Y=Y2−jΔdsin⁡θ+Aml×sin⁡2πjn⁡cos⁡θ.

### 2.2. Lost-Step Processing and Adaptive Step Size Adjustment

*Lost-step* (Figure 1(c)) occurs when the predicted next boundary point cannot be found in a sinusoidal cycle at the sharp position; it indicates that the probe has been away from the desired boundary, so we need to correct the movement direction of probe to correctly track the target boundary. Here, the prediction *θ* is recalculated by adding 90° to the previous *θ*_*i*_  [*θ*_*i*_ = *θ*_*i*−1_ + 90°] and an expanded amplitude (two times of the original amplitude) is used to broaden the search range.

To obtain more abundant contour information and reduce the possibility of the* lost-step* phenomenon, an approximation radius ($\tilde{\rho }$) at* r*_i_ is calculated in real time. It is clear that lower value of $\tilde{\rho }$ represents sharp region, while larger $\tilde{\rho }$ shows smoother regions. Therefore, we can adjust the step size adaptively by detecting the variations of $\tilde{\rho }$. It can be determined by the circumscribed circle of the triangle of three adjacent boundary points (Figure 1(d)):

(7) $$\begin{array}{c} \tilde{\rho }=\frac{abc}{4\sqrt{l\left(l-a\right)\left(l-b\right)\left(l-c\right)}}, \end{array}$$

where *l* = (*a* + *b* + *c*)/2 is the semiperimeter of the triangle defined by the three boundary points* r*_i-3_,* r*_i-2_, and* r*_i-1_, where* a*,* b*, and* c *represent the side length of triangle, respectively. The radius ratio *ρ*_*ratio*_ can be calculated by $\rho_{ratio}=\tilde{\rho }\left(i\right)/\tilde{\rho }(i-1)$, and the step size of the next sinusoidal cycle can be predicted as

(8) $$\begin{array}{c} \Delta_{d}\left(\;i\;\right)=\rho_{ratio}\times \Delta_{d}\left(\;i-1\;\right). \end{array}$$

### 2.3. Local Scanning Based on Boundary Tracking

Unavoidable contaminants near the target edge can cause incorrect boundary points detection or the fitted boundary curve cannot accurately represent the true target boundary, which can have impact correct estimation of the local scan area. To avoid this, the obtained boundary curve is extended before planning the scanning route. However, the problem of equidistant curve has always been a difficult problem in Mathematics because a very small number of special functions of the equidistant curve can be obtained by mathematical methods. Here, we introduce a more appropriate method by combining an analytical method with an improved moving least squares method (MLS) [30] to get the equidistant curve as shown in Figure 1(e). Assuming a known point (*x*, *y*) on a continuous curve with the tangent slope of* k*(*k* = *y*′), the coordinates of the equidistant curve point* M*(*M*′) can be expressed as

(9) xM=x−Tdy′1+y′2,yM=y+Td11+y′2,

where *d* is the offset distance between* M*(*x*_*M*_, *y*_*M*_) and point (*x*,* y*), which is defined by user. *T* = 1 for the equidistant curves upward at the original curve; otherwise, *T* = −1.

A local approximation of the improved MLS method is applied to the original curve for better results by increasing the sampling density of the original data. The local approximation function $\hat{u}(x)$ is expressed as

(10) $$\begin{array}{c} \hat{u}\left(\;x\;\right)=\overset{m}{\underset{i=1}{\sum }}p_{i}\left(\;x\;\right)a_{i}\left(\;x\;\right)=p^{T}\left(\;x\;\right)a\left(\;x\;\right), \end{array}$$

where **p**(*x*) = [*p*_1_(*x*), *p*_2_(*x*),…,*p*_*m*_(*x*)]^T^ is a polynomial basis function and* m* represents the total terms of the basis function. **a**(*x*)=[*a*_1_(*x*), *a*_2_(*x*),…,*a*_*m*_(*x*)]^T^ is the coefficients vector. Thus, a weighted discrete **L**^2^ norm of (10) can be calculated as

(11) J=∑I=1nwx−xI∑i=1mpixaix−uxI2=Pax−uTWxPax−u,

where *n*  (*n* ≤ *N*) is the number of discrete boundary points in the support domain (SD). *N* is the total number of these discrete points and* x*_I_ is the node within the tightly bounded domain of point* x*. **u** = [*u*(*x*_1_), *u*(*x*_2_),…,*u*(*x*_*n*_)]^T^ and **W**(*x*) = diag⁡[*w*_1_(*s*), *w*_2_(*s*),…,*w*_*n*_(*s*)]^T^.

(12) $$\begin{array}{c} P=\left[\begin{array}{ccc} \begin{array}{c} p_{1}\left(x_{1}\right)\\ p_{1}\left(x_{2}\right) \end{array} & \begin{array}{c} p_{2}\left(x_{1}\right)\\ p_{2}\left(x_{2}\right) \end{array} & \begin{array}{cc} \begin{array}{c} \cdots \\ \cdots \end{array} & \begin{array}{c} p_{m}\left(x_{1}\right)\\ p_{m}\left(x_{2}\right) \end{array} \end{array}\\ \vdots & \vdots & \vdots \\ p_{1}\left(x_{n}\right) & p_{2}\left(x_{n}\right) & \begin{array}{cc} \cdots & p_{m}\left(x_{n}\right) \end{array} \end{array}\right]; \end{array}$$

*w*(*x* − *x*_*I*_) is the weight function, which is defined with a cubic spline function:

(13) wis=23−4s2+4s3,s≤0.543−4s+4s2−43s3,0.5<s≤10,s>1,wi′s=−8s+12s2,s≤0.5−4+8s−4s2,0.5<s≤10,s>1;

*s*(*x*) could be defined as

(14) sxi=xval−xi2+yval−yi2max⁡xval−xj2+yval−yj2,s′xi=xval−ximax⁡xval−xj2+yval−yj2xval−xi2+yval−yi2,

where *i*, *j* = 1,2,…, *n*; and

(15) xval=x=xi+1−xikl+xi,yval=xval−xixi+1−xiyi+1−yi+yi,

where *k* represents points between adjacent discrete points* x*_i_ and *x*_*i*+1_; *l* = 1,2,…, *k*.

Equation (11) is solved by using least squares principle:

(16) $$\begin{array}{c} a\left(\;x\;\right)=A^{-1}\left(\;x\;\right)B\left(\;x\;\right)u. \end{array}$$

According to (10) and (16), $\hat{u}(x)$ can be derived:

(17) $$\begin{array}{c} \hat{u}\left(\;x\;\right)=p^{T}\left(\;x\;\right)A^{-1}\left(\;x\;\right)B\left(\;x\;\right)u. \end{array}$$

Thus, its derivative ${\hat{u}}^{\prime }\left(x\right)$ can be expressed as

(18) u^′x=pTx′A−1xBx+pTxA−1x′Bx+pTxA−1xBx′u,

where **A**(*x*) = **P**^T^**W**(*x*)**P**, **B**(*x*) = **P**^T^**W**(*x*), **A**′(*x*) = **P**^T^**W**′(*x*)**P**, **B**′(*x*) = **P**^T^**W**′(*x*), and (**A**^−1^(*x*))′ = −**A**^−1^(*x*)**A**′(*x*)**A**^−1^(*x*).

According to (9) and (18), we can get the approximate fitting function of *M* (*x*_M_,* y*_M_), and the discrete point on equidistance curve can be obtained by downsampling.

Figure 1(f) shows a simulation result of the local scanning algorithm. Slow and fast scan directions (SSD and FSD) of the probe are along* x*-axis and *y*-axis, respectively. *X*_min_ on the equidistant curves is set as the starting position of scan. *Y*_max_ and *Y*_min_ in the fast direction are first calculated and the scanning is initiated in the fast scan direction until *Y*_max_ position is reached. Then scan direction is reversed and the sample is scanned up to *Y*_min_ position. Now the probe moves a unit step length in the slow direction to scan rest area of the target. This process is repeated for each line scan and the imaging is stopped when the tip position on the slow direction reaches *X*_max_.

To sum up, a flowchart showing boundary tracking and scanning method is illustrated in Figure 2.

## 3. Results and Discussions

### 3.1. Experimental Setup

In the preceding sections, we assume that the sample is fixed, while probe is moving. In the experiments, sample is moved, while the probe is fixed. In both cases, the result of the boundary tracking is identical to the only difference in the mode of operation. For AFM experiment, two probes (HQ:NSC18/Al-BS, nominal spring constant: 2.8 N/m, resonance frequency: 75 kHz, MikroMasch and B-lever of HQ:NSC36/No Al, nominal spring constant: 2 N/m, resonance frequency: 130 kHz, MikroMasch) were used in tapping and PeakForce modulation mode, respectively. The sample is fixed on a closed-loop *xyz* scanner (MCL-PDQ375HS, 75 × 75 × 50 *μ*m travel range and 0.15 × 0.15 × 0.1 nm motion resolution, Mad City Labs, Inc.) which is further mounted on an *xy* micropositioning stage for locating the desired position under an optical microscope (20x). An oscillation controller (Dual-OC4, Nanonis GmbH, Switzerland) is used to control the probe dynamics. A data acquisition card (PCI-6363, National Instruments, USA) is utilized for sampling feedback control signal from the lock-in amplifier. A multithread planning and control system has been developed for the feedback control of the *z*-axis and controlling the scanner motion on the *xy*-plane for sample boundary tracking and imaging.

**Figure 3:**
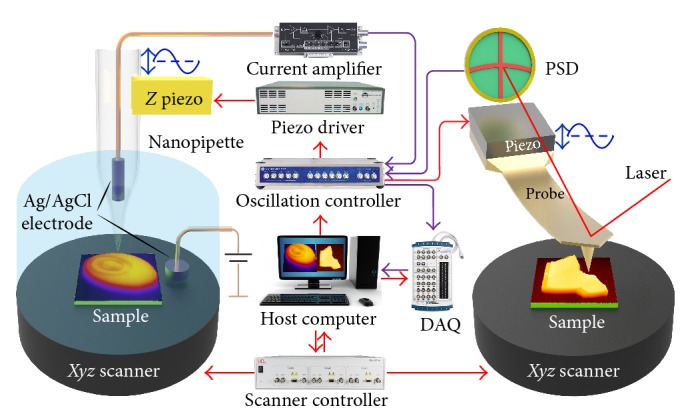
Schematic diagram of integrated SPM system. A sinusoidal signal from an oscillation controller is fed into a piezo driver to drive the piezo for probe oscillation. The modulation distance between the probe and sample surface results in a feedback signal, which is detected by a lock-in amplifier to regulate the tip-sample distance.

A home-built AFM is used with necessary hardware and software modification [31] to construct SICM as shown in Figure 3. A fine tapered borosilicate nanopipette (BF100-58-10, Sutter, USA) with inner tip diameter about 100 nm, filled with 0.1 M KCl aqueous solution, was fabricated through a CO_2_-laser-based micropipette puller (P-2000, Sutter Instrument, Novato, CA). A bias (100 mV) is applied between the Ag/AgCl electrodes inside the pipette and bath. A sinusoidal signal from the oscillation controller is used to drive piezo which sets the nanopipette into vibration. The resulting AC ion current is amplified by a commercial current amplifier (DLPCA-200, FEMTO Messtechnik GmbH, Germany) and fed into the lock-in amplifier. The amplitude of the AC current is applied to the feedback controller to regulate the tip-sample separation against the setpoint.

### 3.2. Boundary Tracking of Microholes

**Figure 4:**
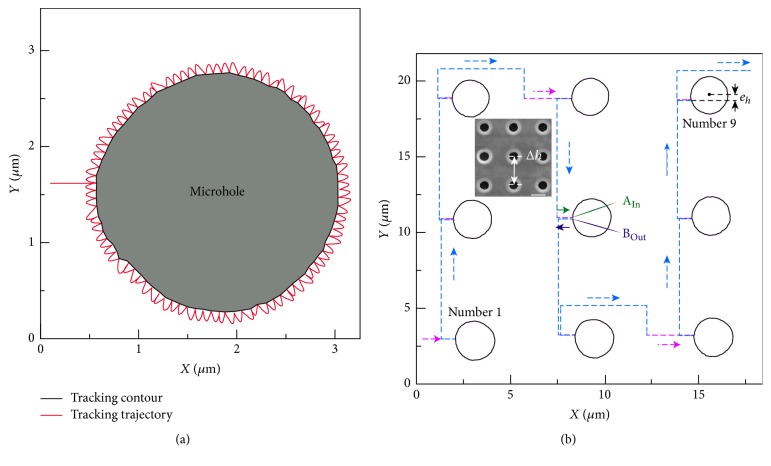
Boundary tracking of microholes. Boundary tracking result of a single microhole (a); microhole array (b). Scale bar: 4 *μ*m.

The probe tracks the outer edge of the microhole without entering into the trench to facilitate the diameter measurement. However, in conventional AFM scan, the tip may be damaged in case of deep microhole with vertical sidewalls. The tracking processes of microhole array can be summarized as follows:

- Calibrate the probe tip position by the SEM image of probe or scan a target under the assistant with optical microscope positioning and image processing. The distance ($\tilde{\Delta_{h}}$) between the centers of two adjacent microholes can be estimated under the optical microscope.
- The tracking procedure is started after the first microhole (the number 1 microhole in Figure 4(b)) is brought near the probe tip with the view field of the optical microscope. The tracking trajectory for the first boundary point of each hole is shown as dashed pink line in Figure 4(b).
- The nanostage is moved along the preset route (moving distance = $e_{h}+\tilde{\Delta_{h}}$; the blue dashed line in Figure 4(b)) to track the next microhole after completing the first microhole. Then a more accurate distance (Δ_*h*_) between the centers of two adjacent holes can be calibrated.
- Follow the microhole array along the predesigned path automatically (as shown in Figure 4(b)).

Figure 4(a) shows the diameter measurement of a microhole by tracking its boundary. The diameter of the microhole measured by the SEM and boundary tracking algorithm are about 2.45 *μ*m and 2.46 *μ*m, respectively. The well agreement in the measurement shows the accuracy of the boundary tracking algorithm. Figure 4(b) shows the boundary tracking of the microhole array, where pink and green dashed line are the tracking trajectories of nanostage; black curves represent the obtained sample contour. Microholes are equally distributed on the *xy*-plane with a center to center distance (Δ_*h*_) of about 8 *μ*m. The average diameter of the microholes measured by boundary tracking algorithm is about 2.46 *μ*m with *σ* of 0.012. Inset shows the SEM image of the microhole array.

Although (Δ_*h*_) can be calibrated in advance, it may not detect any boundary of the next microhole due to the unavoidable inclination of the sample mounting when the forward or retractive position (A_in_ or B_out_ in Figure 4(b), almost same position) is far away from the center of the microhole. Therefore, the position difference (e.g.,* e*_h_ in Figure 4(b)) between B_out_ and the center of the current microhole needs to be compensated to track edges of the microhole array effectively.

### 3.3. Local Scanning of Tapping Mode AFM

**Figure 5:**
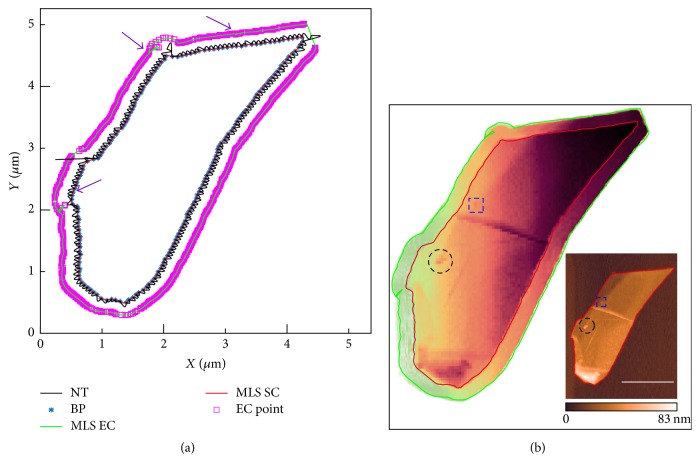
Local raster scanning with AFM. (a) The boundary tracking results on the graphene; (b) AFM topography obtained by the local scanning based on boundary tracking method. Scale bar: 2 *μ*m.

To demonstrate the local scanning performance of the proposed algorithm, a structure of multilayer graphene on silicon substrate was tracked and locally scanned.  Figure 5(a) shows the boundary tracking result that was completed in 6.87 s (tracking velocity: 2.55 *μ*m/s; step length: 10 nm), where blue star and pink squares represent the boundary point (BP) on the sample contour and discrete points on the equidistant curve, respectively. Black, red, and green curve are the nanostage trajectory (NT), fitting sample contour, and equidistant curve (EC), respectively. The sharp corners (indicated by the arrows) appear in numerical results, which are caused by the large variation in the local slope.  Figure 5(b) shows AFM topography obtained by local scanning with the same scanning frequency as conventional raster scanning which was completed in 2.54 min. Inset shows AFM image obtained by the traditional raster scanning (scanning frequency: 0.75 Hz, resolution: 220 × 340 pixels, and step length: 20 nm) which was completed in 4.89 min. As a result, the scan time is saved by 47.97%. It is evident that the edge of the tracked profile (red curve) extracted from Figure 5(a) has a good coincidence with the result image of the raster scan. Both images are matching very well in the small features (dashed box and circle in Figure 5(b)).

### 3.4. Local Scanning with PeakForce Modulation AFM

**Figure 6:**
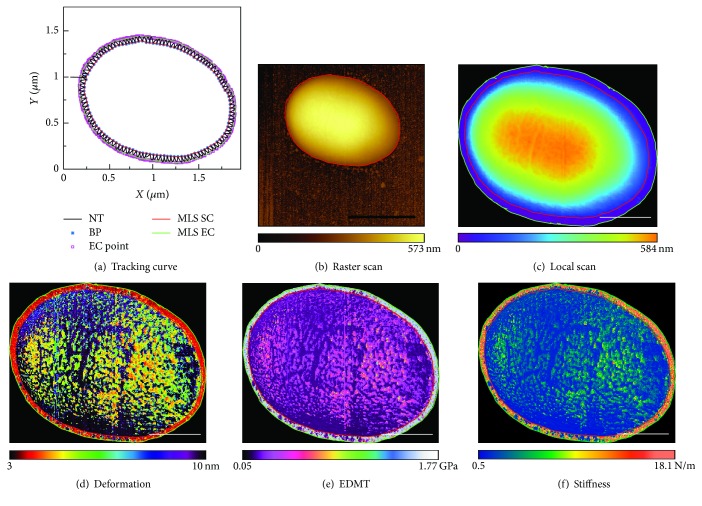
Local raster scanning of an* E. coli *TOP10 cell. (a) Boundary tracking results. (b) Traditional raster scanning. (c) Topography of local raster scanning. (d) Deformation map. (e) Elastic modulus map. (f) Stiffness map. Scale bar (black): 1 *μ*m. Scale bars (white): 500 nm.

*E. coli* TOP10 bacterial cell (freshly dried within 1 hour) was locally scanned by the proposed method to obtain its topography and mechanical properties with PeakForce modulation AFM.  Figure 6(a) shows the boundary tracking result that was completed in 5.57 s (tracking velocity: 1.68 *μ*m/s; step length: 8 nm).  Figure 6(b) shows AFM image obtained by the traditional raster scanning (scanning frequency: 0.75 Hz, resolution: 360 × 350 pixels, and step length: 7 nm) which was completed in 8 min.  Figure 6(c) shows the AFM topography obtained by local scanning with the same scanning speed as conventional raster scanning which was completed in 2.44 min. As a consequence, the scan time is saved by 69.49%. The cell structure can be clearly recognized in the deformation map (Figure 6(d)), elastic modulus map calculated from Derjaguin-Muller-Toporov (DMT) [32] theory (Figure 6(e)), and stiffness map (Figure 6(f)).

### 3.5. Local Scanning with SICM

**Figure 7:**
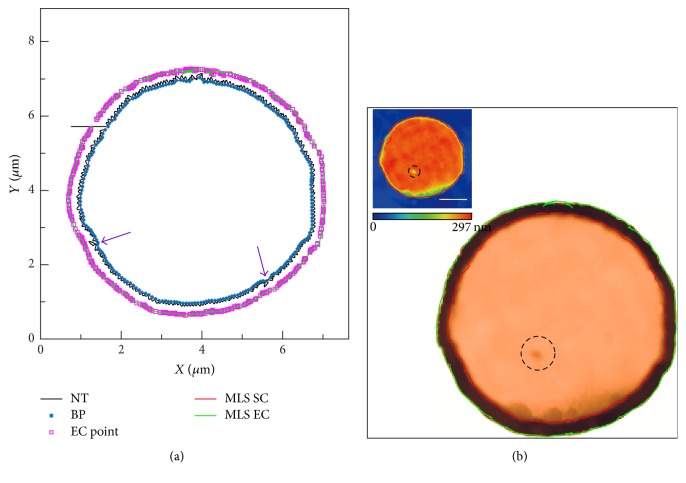
Local raster scanning with AFM. (a) The boundary tracking results on the graphene; (b) AFM topography obtained by the local scanning based on boundary tracking method. Scale bar: 2 *μ*m.

A self-made PDMS sample (Sylgard 184, A/B = 10 : 1, diameter: 6 *μ*m, height: 300 nm) was used to verify the feasibility of the proposed local scanning algorithm with AC-SICM.  Figure 7(a) shows the boundary tracking result that was completed in 10.85 s (tracking velocity: 2.4 *μ*m/s; step length: 10 nm). The sharp corners (indicated by arrows) appear in Figure 7(a), which are caused by the large variation in the local slope.  Figure 7(b) shows AFM topography obtained by local scanning with the same scanning frequency as conventional raster scanning which was completed in 2.69 min (saving about 29.19% of the scan time). Both images are matching very well in the small features (dashed circle in Figure 7(b) and inset image). Inset shows that SICM image is obtained by the traditional raster scanning (scanning frequency: 1 Hz, resolution: 228 × 240 pixels, and step length: 30 nm) which was completed in 3.8 min. The red curve shows that the boundary curve extracted from the boundary tracking data has a good coincidence with 2D SICM image.

## 4. Conclusions

A boundary tracking and local scanning method is developed for fast scanning of the region of interest. The prior boundary information of the target is recorded with boundary tracking method in the first pass; the local scanning is performed in a second pass for only mapping the region that is confined by the first pass. Moreover, the boundary tracking algorithm can be separately used for the fast dimension metrology, and the reliability and practicality of this algorithm are validated by fast tracking of single/multimicroholes. Experimental results demonstrate that the proposed method is faster than traditional raster scanning by lots of the saving time. The applicability and versatility of the method are confirmed by using SICM and AFM experiments. The developed local scanning method would show great potential in the field of morphology measurement and the method is expected to be widely used in scanning probe microscopy.
